# Severe Thymic Atrophy in a Mouse Model of Skin Inflammation Accounts for Impaired TNFR1 Signaling

**DOI:** 10.1371/journal.pone.0047321

**Published:** 2012-10-11

**Authors:** Nathalie Belhacéne, Parvati Gamas, Diogo Gonçalvès, Marie Jacquin, Marie Beneteau, Arnaud Jacquel, Pascal Colosetti, Jean-Ehrland Ricci, Abdellilah Wakkach, Patrick Auberger, Sandrine Marchetti

**Affiliations:** 1 Inserm, UMR 1065, Centre Méditerranéen de Médecine Moléculaire, Team «Cell death, differentiation, inflammation and cancer», Nice, France; 2 Inserm, UMR 1065, Centre Méditerranéen de Médecine Moléculaire, Team «Metabolic control of cell deaths», Nice, France; 3 CNRS, FRE 3472, LP2M, Nice, France; 4 Université de Nice Sophia Antipolis, Faculté de Médecine, Nice, France; 5 Equipe labellisée par la Ligue Nationale Contre le Cancer 2011–2013, Paris, France; Cedars-Sinai Medical Center, United States of America

## Abstract

Transgenic mice expressing the caspase-cleaved form of the tyrosine kinase Lyn (LynΔN) develop a TNFα-dependent skin disease that accurately recapitulates human psoriasis. Participation of lymphocytes in this disease was confirmed by backcrossing LynΔN mice on a Rag-1 deficient background. The present study was therefore conducted to analyze whether modification of lymphocyte homeostasis does occur and participate in the phenotype of LynΔN mice. We show here that LynΔN mice consistently exhibit thymic atrophy that correlates with both a net decrease in the CD4+/CD8+ Double Positive (DP) and an increase in Single Positive (SP) thymocyte sub-populations, but also display an increase of splenic mature B cell. Interestingly, a normal immune phenotype was rescued in a TNFR1 deficient background. Finally, none of these immune alterations was detected in newborn mice before the onset of inflammation. Therefore, we conclude that chronic inflammation can induce thymic atrophy and perturb spleen homeostasis in LynΔN mice through the increased production of TNFα, LTß and TNFR1 signaling.

## Introduction

Tumor necrosis factor (TNF), Lymphotoxin-α (LTα) and Lymphotoxin-ß (LTß) are structurally related molecules that belong to the TNF superfamily of pro-inflammatory cytokines [1], [2]. TNF and LTα are secreted as homotrimers that can bind and activate both the TNFR1 and TNFR2 receptors [3]. LTß and LTα bind together to form a membrane-anchored heterotrimer composed of two LTß and one LTα (LTß2-LTα1) proteins that triggers specifically the LTß receptor (LTßR) [2]. Signaling through the LTßR is essential for the Peyer’s patches and lymph node development and for the maintenance of the overall organization of peripheral lymphoid organs in adult animals [4], [5]. LTα and LTß deficient mice displayed abnormal spleen homeostasis that can be complemented with expression of TNFα [6].

Thymic involution can occur in different circumstances including stress, infection or acute inflammation [7], [8]. However, these modifications are most of the time reversible and a normal thymus size and cellularity are generally restored when the causative agent is eliminated. Both TNF and LTs are upregulated by inflammatory stimuli and through binding to their specific receptors can regulate thymic atrophy [5], [9], [10]. Accordingly, a recent study by Liepinsh et al. reported that elevated homeostatic expression of the genes encoded by the TNF/Lymphotoxin locus in a transgenic mice model results in thymic atrophy [9] which was strictly related to the level of expression of both TNFα and LTß. In addition, transgenic overexpression of LIGHT, another TNF-related ligand in T cells results in drastic thymus atrophy, associated with increased apoptosis and reduced numbers of DP T cells [11].

Lyn is a non-receptor tyrosine kinase of the Src family that participates in the regulation of many cellular functions of pluricellular organisms, including proliferation, differentiation and cell death [12], [13]. We have established that, during apoptosis, Lyn is cleaved by executioner caspases [14], [15]. We recently reported that transgenic mice expressing the caspase-cleaved form of the tyrosine kinase Lyn (LynΔN transgenic mice) developed a severe cutaneous inflammatory disease recapitulating accurately human psoriasis [16]. LynΔN transgenic mice exhibited also elevated levels of pro-inflammatory cytokines including TNFα, LTß and IL-1ß. The importance of the increased production of LTß and TNFα in these mice was further supported by the restoration of a completely normal phenotype in a TNFR1 deficient background.

In the present study, we therefore characterized the immune phenotype of LynΔN mice. We found that LynΔN mice exhibited a severe thymic atrophy characterized by a massive depletion of the CD4^+^/CD8^+^ (DP) thymocyte sub-population, associated with an increase in CD19/B220 mature B cell in the spleen. These immune defects observed in LynΔN mice were totally abolished in a TNFR1 deficient background suggesting the involvement of TNFα and possibly LTß in the impairment of thymocyte development and spleen homeostasis. We propose that chronic inflammation can induce thymic atrophy in LynΔN mice through increase production of TNFα and LTß. In addition, alteration of immune cell maturation could participate to the psoriasis-like disease developed by these mice.

**Figure 1:**
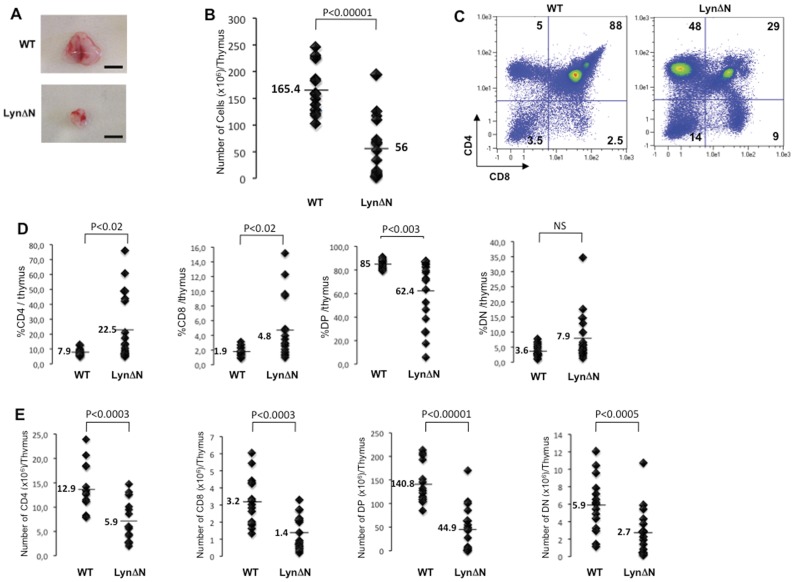
Abnormal thymocyte maturation in LynΔN transgenic mice. **A)** Representative thymi from 2-week old control and LynΔN mice. Bare scale : 0.5 cm. **B)** Total thymocyte numbers were determined. Lines indicate the mean, and each symbol represents one individual mouse. **C)** Total thymocytes from LynΔN and control mice were analyzed by flow cytometry for CD4 and CD8 expression, as shown in this representative flow cytometry profile. **D)** Proportion and **E)** number of all thymocyte subsets of 2-week old control and LynΔN mice were quantified; Lines indicate the mean, and each symbol represents one individual mouse**.** For these experiments, n = 15 for WT and n = 17 for LynΔN mice. P value, Student *t* test for unpaired data.

## Results

### Thymic Atrophy and Altered Thymocyte Maturation in LynΔN Transgenic Mice

We reported previously that LynΔN mice exhibited very early after birth a TNFα-dependent cutaneous syndrome that recapitulates accurately human psoriasis [16]. As psoriasis is a T cell dependent disease [17], we investigated whether thymocyte selection and maturation were altered in 2-week old LynΔN mice. Interestingly, thymi from LynΔN mice were consistently smaller than the one of wild-type mice (Figure 1A). Accordingly, there was a three-fold reduction in the total number of thymocytes in LynΔN mice (Figure 1B). Flow cytometry analysis of the four main thymocyte subpopulations showed a drastic decrease in the proportion of CD4^+^/CD8^+^ DP thymocytes in favour of the SP thymocyte population (Figure 1C and 1D). No significant difference was observed for the DN population (CD4−/CD8−). By contrast, the LynΔN transgenic mice exhibited fewer number of cells in each thymocyte subsets compared to control mice (Figure 1E). In this line, compared to age-matched littermate control mice, SP cells (CD4+ or CD8+) and DP cells (CD4+/CD8+) were reduced by 2-fold and 3-fold respectively. The drastic loss of thymus cellularity was quite general over the thymocyte subsets but seems to mainly affect the DP population.

**Figure 2:**
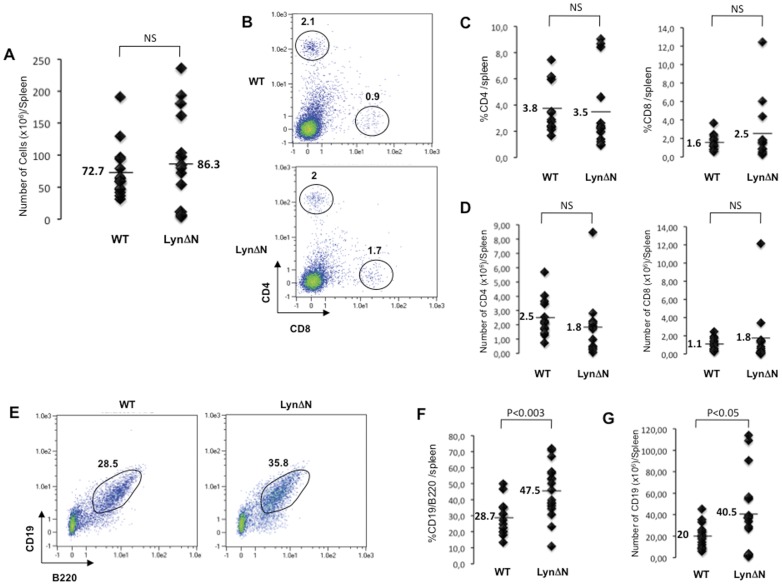
Normal splenic T cell but abnormal B cell distribution in LynΔN transgenic mice. **A)** Total splenocyte numbers were determined. Lines indicate the mean, and each symbol represents one individual mouse. **B)** Splenic T cells from LynΔN and control mice were analyzed by flow cytometry (CD4 and CD8 expression), as shown in this representative flow cytometry profile. **C)** Proportion and **D)** cell numbers of CD4+ and CD8+ splenic T cell of 2-week old control and LynΔN mice were quantified; Lines indicated the mean, and each dot represents one individual mice. For these experiments, n = 15 for WT and n = 17 for LynΔN mice. P value, Student *t* test for unpaired data. **E)** Splenic B cells from LynΔN and control mice were analyzed by flow cytometry (CD19 and B220 expression), as shown in this representative flow cytometry profile. **F)** Proportion and **G)** number of CD19+/B220+ splenic B cell of 2-week old control and LynΔN mice were quantified; Lines indicated the mean, and each dot represents one individual mice. For these experiments, n = 15 for WT and n = 17 for LynΔN mice. P value, Student *t* test for unpaired data.

### Abnormal B Cell Distribution in LynΔN Transgenic Mice

We next analyzed the splenic phenotype of LynΔN mice. Although the total number of splenocytes was slightly higher in LynΔN mice compared to their wild-type counterpart, there were considerable variations in the total number of spleen cells in individual LynΔN mice (Figure 2A). To gain insights into the splenic phenotype of LynΔN mice, we performed flow cytometry experiments in order to decipher the T and B cell distribution of LynΔN and control spleen. Analysis of splenic CD4+ and CD8+ T cell showed no significant difference either in proportion (Figure 2B and 2C) or in cell number (Figure 2D). In contrast, LynΔN mice had a 1.7-fold increase in proportion (Figure 2E and 2F) and a 2-fold increased number (Figure 2G) of CD19^+^/B220^+^ mature B cells compared to wild-type mice. These results indicate that LynΔN mice displayed a perturbed spleen homeostasis.

**Figure 3:**
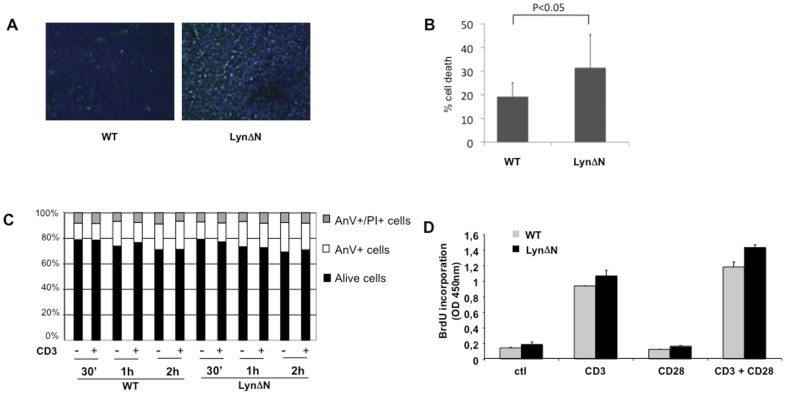
TCR dependent proliferation and apoptosis are unaltered in total thymocytes of LynΔN mice. **A)** Apoptosis index determined by TUNEL staining (green staining) was analyzed onto frozen thymi sections of 2-week old LynΔN and control mice. **B)** AnnexinV positive cells were analyzed onto freshly isolated thymocytes from control and LynΔN transgenic mice (n = 6 in each group) by flow cytometry. **C)** Total thymocytes from LynΔN and control mice were stimulated or not with 10 µg/ml of plate-coated anti-CD3 for the indicated time periods and AnnexinV+/PI+ dead cells were determined by flow cytometry. **D)** Total thymocytes from LynΔN and control mice were stimulated with 10 µg/ml of plate-coated anti-CD3 plus soluble anti-CD28 (2 µg/ml) mAbs for 72 h. 10 µM BrdU was added for 16 hours, and proliferation was measured by BrdU incorporation. Results are expressed as mean ± SD. Data are representative of at least 3 independent experiments.

### The Massive Thymus Atrophy Observed in LynΔN Transgenic Mice is Linked to Increase Thymocyte Cell Death

As the massive thymus atrophy observed in LynΔN transgenic mice could be due to increased thymocyte cell death, we performed TUNEL analysis. As shown in Figure 3A, high apoptosis index was observed in LynΔN thymi as compared to control, indicating that the thymus atrophy is likely due to a rise in cell death. In addition, annexin-V analysis of freshly isolated thymocyte confirmed the increased cell death of thymocytes derived from LynΔN transgenic mice (figure 3B). Finally, all thymocyte subpopulations seemed to be equally affected (data not shown).

**Figure 4:**
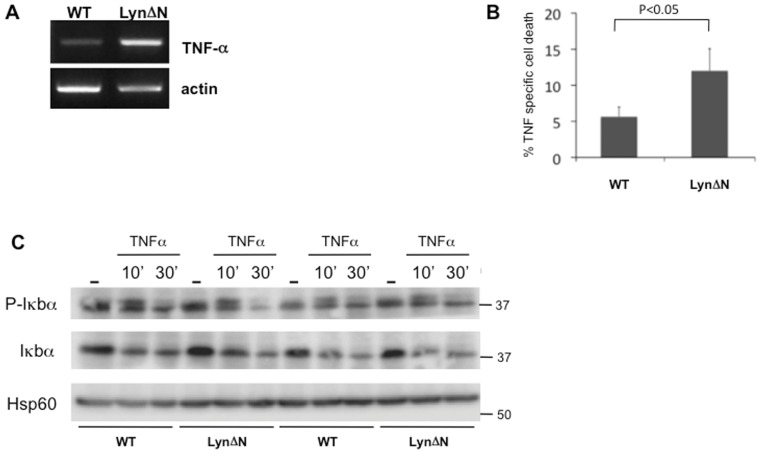
TNF specific cell death is increased in LynΔN thymocytes. **A)** Semi-quantitative RT-PCR analysis of TNFα and actin using RNA isolated from 2-week-old LynΔN and control thymi. **B)** Freshly isolated thymocytes from control or LynΔN mice were left untreated or incubated with either TNFα (50 ng/ml) for 18 h. Then, AnnexinV positive cells were determined by flow cytometry. Results are expressed as the percentage of TNF specific cell death (percentage of AnV+ cells in TNF stimulated condition – percentage of AnV+ cells in control condition). For this experiment, n = 3 for WT and n = 4 for LynΔN mice. P value, Student *t* test for unpaired data. **C)** Freshly isolated thymocytes from control or LynΔN mice were left untreated or incubated with either TNFα (50 ng/ml) for 10 and 30 minutes. Degradation and phosphorylation of IκBα was assayed by western blot analysis of total cell extracts. Hsp60 was used as a loading control.

Thus, we further investigated whether LynΔN thymocytes could be more sensitive to apoptosis ex-vivo. For that purpose, freshly isolated thymocytes from WT and LynΔN mice were incubated for various periods of time with an anti-CD3-mAb. Figure 3C showed that over the kinetic and even after 6 h of stimulation (data not shown), the percentage of annexin V positive cells were similar in WT and LynΔN thymocytes indicating that neither apoptosis-induced cell death (AICD) nor spontaneous apoptosis was affected in thymocytes from transgenic mice. Furthermore, to investigate whether the decreased number of thymocytes in LynΔN accounts for impaired proliferation, freshly isolated thymocytes were incubated for 48 hours in the presence of an anti-CD3-mAb alone or in combination with an anti-CD28-mAb and further incubated for 48 hours with BrdU. As shown on Figure 3C, there was no modification of the proliferation index of thymocytes from control or LynΔN mice. These results suggest that LynΔN mice exhibit no signaling defect following T cell receptor engagement. Altogether, our results show that the decrease in thymocyte number was neither due to an increase of thymocyte apoptosis nor to an impairment of proliferation, suggesting a normal activation in response to TCR engagement, and supporting the conclusion that SP thymocytes from LynΔN are functional.

**Figure 5:**
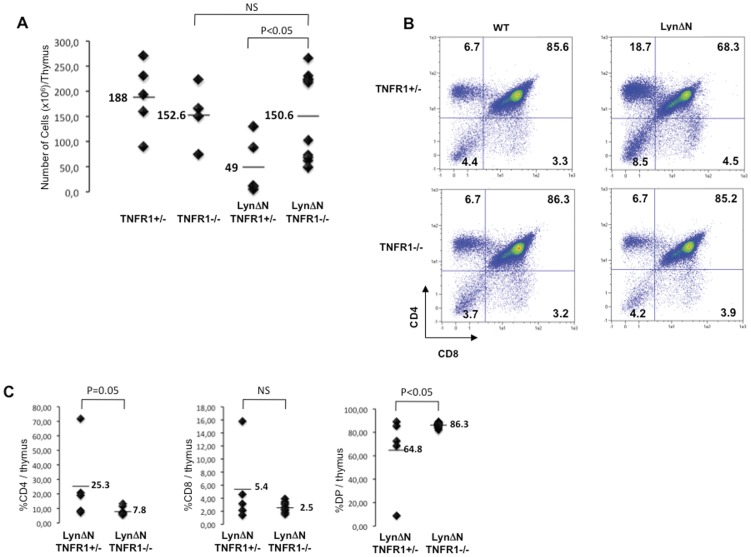
Thymic alteration in LynΔN mice is dependent on TNFR1 signaling. **A)** Total thymocyte numbers from LynΔN and control mice in TNFR1+/− or TNFR1−/− background were determined. Lines indicate the mean, and each symbol represents one individual mouse. **B)** Total thymocytes from LynΔN and control mice in TNFR1+/− or TNFR1−/− background were analyzed by flow cytometry for CD4 and CD8 expression, as shown in this representative flow cytometry profile. **C)** Proportion of all thymocyte subsets was quantified for LynΔN/TNFR1+/− and LynΔN/TNFR1−/− mice; each dot represents one individual mice. For these experiments, n = 5 for TNFR1+/−, TNFR1−/−, LynΔN/TNFR1+/− and n = 10 for LynΔN/TNFR1−/−. P value, Student *t* test for unpaired data.

We have previously identified that LynΔN transgenic mice displayed an increase expression of pro-inflammatory cytokine, such as TNFα [16]. As elevated homeostatic expression of the genes encoded by the TNF/Lymphotoxin locus in mice results in thymic atrophy [9], we first checked that TNFα mRNA expression was increased in LynΔN mice thymi (Figure 4A). We further analyzed thymocyte cell death upon TNFα treatment ex-vivo [18]. Figure 4B shows that thymocyte isolated from LynΔN transgenic mice are more sensitive to TNFα induced cell death as compared to control cell, although early events (IκBα phosphorylation and degradation) induced by TNFR activation were induced to the same extent in both control and LynΔN thymocytes (figure 4C).

**Figure 6:**
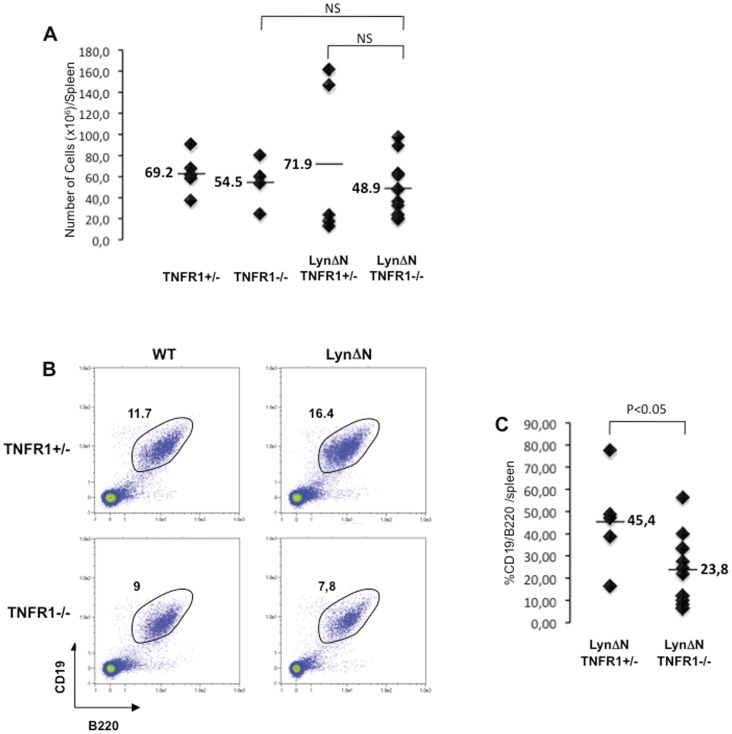
Splenic alterations in LynΔN mice are dependent on TNFR1 signaling. **A)** Total splenocyte numbers from LynΔN and control mice in TNFR1+/− or TNFR1−/− background were determined. Lines indicate the mean, and each symbol represents one individual mouse. **B)** Splenic B cells from LynΔN and control mice in TNFR1+/− or TNFR1−/− background were analyzed by flow cytometry (CD19 and B220 expression), as shown in this representative flow cytometry profile. **C)** Proportion of CD19/B220 splenic B cell was quantified for LynΔN/TNFR1+/− and LynΔN/TNFR1−/− mice; each dot represents one individual mice. For these experiments, n = 5 for TNFR1+/−, TNFR1−/−, LynΔN/TNFR1+/− and n = 10 for LynΔN/TNFR1−/−. P value, Student *t* test for unpaired data.

### Thymic and Splenic Alterations in LynΔN are Dependent on TNFR1 Signaling

As cytokines of the TNF family are increased in LynΔN transgenic mice ([16] and figure 4A), we decided to investigate whether this was also the case for the immune cell alterations observed in these mice. As shown on Figure 5A, TNFR1^+/−^ and TNFR1^−/−^ mice had an equivalent number of thymocytes, while it was reduced in LynΔN/TNFR1^+/−^ mice. Importantly, a normal thymocyte number was restored in a LynΔN/TNFR1^−/−^ background (Figure 5A) and was associated with a normal distribution (Figure 5B and 5C) and cell number (data not shown) of the four above-mentioned thymocyte subpopulations, as compared to LynΔN/TNFR1^+/−^.

**Figure 7:**
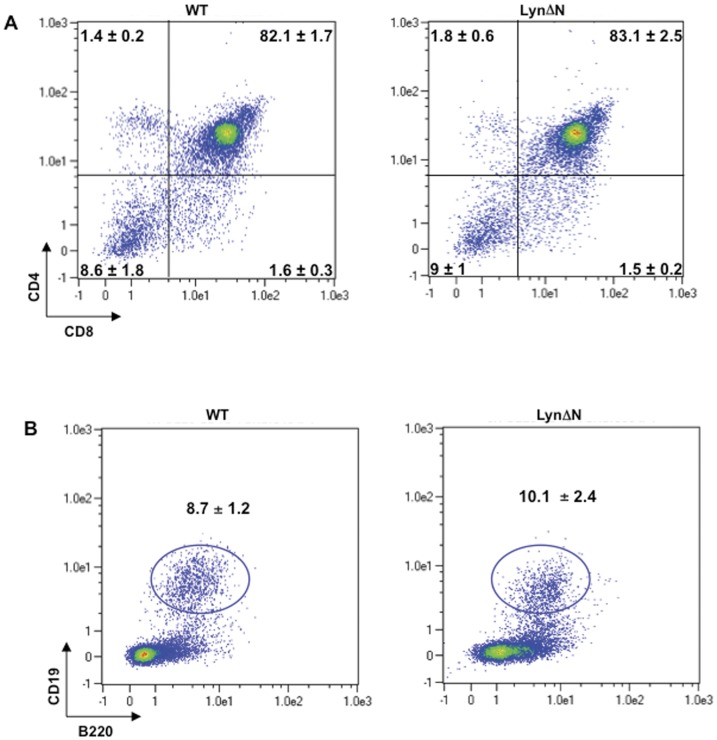
Newborn LynΔN mice displayed normal thymocyte and splenic B cell populations. **A)** Total thymocytes from LynΔN and control mice were analyzed by flow cytometry for CD4 and CD8 expression. **B)** Splenic B cells from LynΔN and control mice were analyzed by flow cytometry (CD19 and B220 expression). Data are the mean ± SD; n = 4 for WT and n = 6 for LynΔN mice.

In the same line, a normal and homogeneous number of splenic cells was found in the spleen of LynΔN/TNFR1^−/−^ mice as compared to LynΔN/TNFR1^+/−^ (Figure 6A). The increase in mature B cells detected in the spleen of LynΔN mice was also lost in a TNFR1 deficient background (Figure 6B and 6C).

The psoriasis-like syndrome displayed by LynΔN mice is detected five days after birth [16]. As the immune cell alterations of LynΔN mice are dependent on TNFR1 signaling, it should not arise in newborn LynΔN mice before the onset of the disease [16]. Conversely to the situation observed 2 week after birth, total thymocyte and splenocyte number was the same in newborn control and LynΔN transgenic mice (data not shown). Accordingly, flow cytometry analysis of thymocytes from newborn LynΔN mice showed a normal distribution of the four main T cell sub-populations compared to wild-type mice (Figure 7A). Moreover, the same percentage of mature CD19+/B220+ B cells was detected in the spleen of control and LynΔN newborn mice (Figure 7B), even though 3 times less mature B cells were detected in newborn mice compared with 2-week-old mice (Figure 2 and 7). Taken together our findings strongly suggest that the immune phenotype of LynΔN mice is consecutive to the onset of the inflammatory disease displayed by LynΔN mice and not due to intrinsic defect.

## Discussion

In the present study, we demonstrate that TNF-dependent chronic inflammation developed by LynΔN transgenic mice has a dramatic impact onto hematopoietic homeostasis. Thus, we established that these mice displayed a severe thymic atrophy and an increase in spleen mature B cells, both defects that are totally rescued in a TNFR1 deficient background. This finding strongly suggests that TNF and/or LT control thymus cellularity and spleen homeostasis in our mice model. This observation is in agreement with several recent reports from the literature regarding thymus atrophy in various conditions. Indeed, Liepshin et al. recently reported that transgenic expression of the TNF/lymphotoxin cytokine locus in mice triggers accelerated thymus atrophy [9]. In this study these authors also showed, using reciprocal bone marrow transplantation that both LTßR and TNFR1 signaling participated in accelerated thymus degeneration. They concluded that chronic infection or inflammation could promote thymic atrophy by up-regulation of both LT and TNF production. All together, these findings are in very good agreement with our own data showing that chronic inflammation in LynΔN mice can promote thymus atrophy. It remains however to determine whether the increased in the CD4+ and CD8+ SP subpopulation reflects an increase capacity of LynΔN thymocytes to maturate. Moreover, an alternative possibility will be that more SP thymocytes can exit from the thymus to the lymph nodes or the peripheral blood. In addition, the increased proportion of CD4+ and CD8+ T-cells in LynΔN mice might account at least in part for the T cell infiltration consistently found in the skin of LynΔN mice [16], a situation that also occurs in the lesions of psoriatic patients which exhibit massive infiltration by activated T lymphocytes [17].

We also observed a large number of apoptotic cell death in LynΔN thymus as compared to age-matched control mice. Our in vitro data indicated that the increased apoptosis observed in LynΔN thymus was not due to a cell-autonomous defect of LynΔN thymocytes but rather to a paracrine response of these cells to cytokines secreted in response to inflammation. Moreover, LynΔN thymocyte seemed to be more sensitive to TNFα-induced cell death than control thymocytes. This observation is in agreement with our previous data showing that LynΔN could inhibit NFκB signaling [16]. Mice deficient for several members of the NFκB signaling pathway did not display abnormal thymocyte or B cell differentiation [19]. By contrast, mice deficient for IKK2, in which NFκB signaling is impaired, displayed thymus atrophy and an increase in thymocyte cell death induced by TNFα [20] . Abolition of TNFR1 signaling in these mice restore a normal thymocyte distribution, indicating that NFκB signaling is essential for protecting T cells from TNFα-induced apoptosis. Thus, one can postulate that increased TNF expression into LynΔN thymus could induce apoptosis of thymocytes, that could reflect NFκB inhibition due to LynΔN expression, as previously reported [16]. However, we cannot rule out the implication of other mechanisms controlling thymocyte cell death in LynΔN transgenic mice.

Transgenic mice in which TNF/LT signaling is abolished due to the expression of a TNF receptor p60-Fc develop different abnormalities of secondary lymphoid organs and more precisely displayed a diminution of B220+ B cells into the spleen [21], indicating that TNF and LT are crucial for normal peripheral development. In our mouse model of TNF-dependent chronic inflammation we also observed an increase in splenic B cells. All together these observations confirmed that TNF and LT are important regulator of spleen homeostasis and that modulation of their expression induced severe defect in immune homeostasis that can have important impact on immune responses.

In conclusion, we have developed a transgenic mice model that displayed an inflammatory syndrome dependent on TNFR1 signaling with important repercussion on skin and immune homeostasis.

## Materials and Methods

### Transgenic and KO Mice

C57Bl6 LynΔN transgenic mice have been previously described [16]. LynΔN were backcrossed with TNFR1 deficient mice (Jackson Laboratory). Animal studies were approved by the Institutional Animal Care and Use Committee of the Centre Méditerranéen de Médecine Moléculaire (INSERM UMR 1065).

### Antibodies and Reagents

CD4, CD8, CD44, CD25, CD19 and B220 were from BD bioscience pharmingen. CD3 antibody was purified from hybridoma (clone 2C11). CD28 antibody was from SouthernBiotech (clone 37.51). Rabbit anti-IκBα (sc-203) and goat anti-hsp60 (sc- were purchased from Santa-Cruz. Anti-phospho IkBα and anti-rabbit HRP were purchased from Cell signaling. Anti-goat-HRP was from Dako cytomaton. Mouse TNFα was from peprotech.

### Preparation of Thymocytes and Splenocytes

For the preparation of cells from thymus and spleen, the organs were removed from individual mice and passed through a nylon membrane (70 µM porosity) to obtain single cell suspensions in RPMI 1640 medium (with 10% fetal calf serum, 50 µM ß-mercaptoethanol, 100 µg/ml each of penicillin and streptomycin, 2 mM glutamine).

### Flow Cytometry Analysis

Thymocytes or splenocytes (10^6^ cells) were labeled with the appropriate fluorochrome-conjugated antibodies. Acquisition was performed in a MACSquant cytometer (Miltenyi) and analysed with MACquant software.

### RT-PCR Analysis

Tissues were harvested and incubated in RNAlater (Ambion) before RNA extraction. Total RNAs were isolated using Trizol Reagent (Invitrogen) after tissue homogenization with polytron. The supernatant was cleared by centrifugation, precipitated with isopropanol, and resuspended in RNAse and DNAse-free water.

For RT-PCR, total RNA (1 µg) was reverse transcribed using the SuperScript II reverse transcriptase (Invitrogen) following the manufacturer’s instructions in a 40 µl final volume. cDNAs (2 µl) were amplified using 1 U of Taq polymerase (New England Biolabs, Ipswich, MA, USA) in a final volume of 25 µl buffer containing 1.5 mM MgCl2, 0.2 mM deoxynucleoside triphosphate (dNTP) and 0.5 µM of the primers for TNFα [16].

### BrDU Incorporation

For proliferation, 1.5×10^5^ thymocytes were seeded in 96-well flat-bottom tissue culture plates with 10 µg/ml of plate-coated anti-CD3 plus soluble anti-CD28 (2 µg/ml) mAbs. After 72 h, cells were pulsed with 10 µM BrdU for 16 hours. Incorporated BrdU was quantified using the Cell Proliferation ELISA colorimetric BrdU kit (Roche).

### Annexin V/PI Staining

After treatment, cells were resuspended in 200 µl of buffer (150 mM NaCl, 10 mM Hepes, 5 mM KCl, 1 mM MgCl2, 1.8 mM CaCl2) and incubated with recombinant Annexin V-fluorescein isothiocyanate for 10 min at room temperature. A volume of 0.5 µg/ml of propidium iodide was then added, and samples were analyzed immediately by flow cytometry using a FACScan (Becton Dickinson, Franklin Lake, NJ, USA). Percentage of apoptotic cells corresponds to Annexin V-positive cells and Annexin V/propidium iodide double positive cells.

### TUNEL Staining

Tissue sections (6 µm) of frozen thymi were analyzed for in situ cell death according to manufacturer guidelines (Roche). Preparations were mounted in fluoromount (Southern Biotech) and images were acquired with Leica microscope and LAF6000 software.

### Immunoblotting

After stimulation, cells were lysed in a buffer containing 50 mM Tris pH 7.5, 100 mM NaCl, 5 mM EDTA, 10 mM NaF, 10 mM Na3VO4, 1 mM PMSF, 1 mM leupeptin, 20 µg/ml aprotinin and 1% Triton X-100. Proteins were separated by SDS-PAGE and transferred onto PVDF membrane (Immobilon-P, Millipore). After blocking nonspecific binding sites, the membranes were incubated with specific antibodies. The membranes were washed and incubated further with horseradish peroxidase conjugated antibody. Immunoblots were revealed by autoradiography using the enhanced chemiluminescence detection kit (Pierce).
